# Temperature Experiment and Parameter Optimization of Cemented Carbide Tool in Milling 508III Steel

**DOI:** 10.3390/ma16072833

**Published:** 2023-04-02

**Authors:** Yaonan Cheng, Xiaoyu Gai, Rui Guan, Yingbo Jin, Mengda Lu

**Affiliations:** School of Mechanical Engineering, Harbin University of Science and Technology, Harbin 150080, China

**Keywords:** temperature experiment, parameter optimization, response surface method, cemented carbide tool, 508IIIsteel

## Abstract

In machining 508III steel, the cemented carbide tool is subjected to a strong periodic thermal load impact, leading to serious tool-chip adhesion and shortening the tool life. Considering the influence of cutting parameters on temperature, temperature experiments and finite element (FE) simulations were carried out based on Box-Behnken experimental design criteria in the response surface method (RSM). Based on the experimental results, A second-order polynomial regression prediction model for temperature was constructed as the optimization objective function based on RSM. A temperature prediction model based on GA-SVM was established to predict temperature change. Taking cutting temperature and efficiency as evaluation indicators, the elitist nondominated sorting genetic algorithm was used to optimize cutting parameters. These findings may be important for the tool life improvement and reasonable parameter selection.

## 1. Introduction

The water chamber head is an important part of the steam generator of the nuclear island. The material is high-intensity 508III steel. The blank of the workpiece is forged as a whole, and the surface to be processed is poor [1,2]. The cemented carbide tool usually was adopted. Due to the high strength, good low-temperature impact toughness, and low non-ductility transition temperature of the 508III steel, it causes a higher cutting temperature in the cutting. Due to the large thermal load shock and the difficult machining of 508III steel, the phenomenon of tool-chip bonding often occurs during machining, which shortens the tool life and seriously affects the machining efficiency and machining quality [3]. Aiming at the above problems, it is necessary to conduct an in-depth study of the cutting temperature during the process of milling 508III steel with the cemented carbide tool.

The cutting temperature is one of the primary reasons for tool failure in machining. Periodic and thermal shocks can cause cracks easily in the tool material, leading to its failure and affecting the tool’s life seriously. Over the years, many scholars at home and abroad have studied cutting temperature from different angles. Ueda et al. [4] measured the cutting temperature of Ti-6Al-4V and Inconel718 material using a self-developed optical fiber two-color pyrometer. Patru et al. [5] used an infrared thermometer to measure the temperature while milling the aluminum alloy. Bhirud et al. [6] measured the temperature during end-milling aluminum alloys using a K-type thermocouple. Sato et al. [7] used an infrared radiation pyrometer to measure the temperature of end-milling titanium alloys. Yang et al. [8] studied the temperature of turning titanium alloys based on FE simulation and found that the influence degree was *v_c_* > *a_p_* >*γ_0_* > *α_0_* > *r*. Gao et al. [9] established a thermal model for predicting the temperature field of turning tools based on modulation-assisted. Liu et al. [10] proposed a method of transmitting temperature signals through wireless communication to measure the temperature. Bi et al. [11] established the FE model of temperature based on the given heat source method. Geng et al. [12] conducted a temperature test on titanium alloy by sandwich semi-artificial thermocouple method.

For many years, parameter optimization has been a hot issue with which scholars are concerned. Many scholars have studied parameter optimization based on different methods with different response variables as optimization objectives. George et al. [13] optimized the parameters of stainless steel based on the Taguchi method, taking the surface roughness as the target. Nguyen et al. [14] adopted the Taguchi method to optimize cutting parameters with the objective of optimizing surface roughness and productivity and obtaining the best parameter combination. Santhanakrishnan et al. [15] used GA to optimize the processing parameters of aluminum alloy with the optimization goal of temperature rise. Librantz et al. [16] took production and machining efficiency as the optimization goal, and GA was used to optimize the milling parameters. Zain et al. [17] used the combination of GA and neural networks to predict and optimize the surface roughness of milling titanium alloy. Tao et al. [18] used cutting simulation and GA to optimize the parameters of superalloy and obtained the optimal processing parameters. Chen et al. [19] used the extended non-dominated sorting genetic algorithm to optimize milling parameters with production efficiency and tool life consumption as the target.

Relevant scholars have laid a certain foundation for the research of temperature experiments and parameter optimization in machining, and most of the research mainly focused on materials such as titanium alloy, aluminum alloy, and carbon steel. However, there were relatively few studies on temperature experiments and parameters of heavy-milling 508III steel. Therefore, through the combination of FE simulation, GA-SVM prediction, and multi-objective optimization methods, this paper made an in-depth study of the tool temperature experiment and cutting parameter optimization in milling 508III steel. The findings have important theoretical and practical significance for the reasonable selection of machining parameters, the extension of tool life, and the improvement of machining efficiency and quality.

## 2. Temperature Experiment and FE Analysis of Milling 508III Steel

### 2.1. Temperature Experimental Protocol and Measurement Method Design

**Figure 1:**
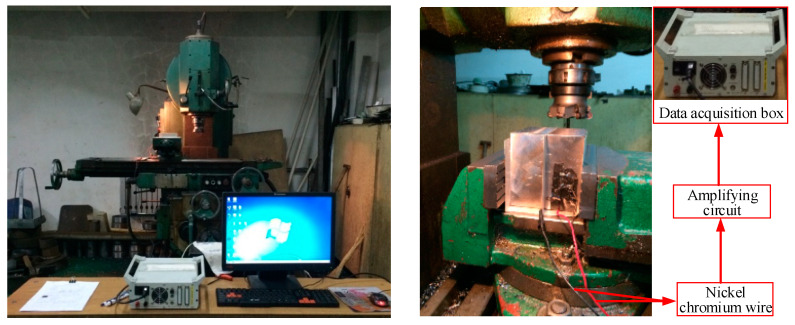
Field diagram of the milling temperature experiment.

The milling experiment was carried out on the vertical lifting table milling machine. The cutting temperature was measured by the method of the wire-clamping semi-artificial thermocouple. A nickel-chromium wire was clamped in the middle of the workpiece, which was divided into two parts as the hot end. The other thermocouple was welded at the bottom of the workpiece as the cold end. When the thermocouple wire was cut off, the semi-artificial thermocouple converted the temperature difference between the hot end and the cold end into a voltage signal and collected voltage data through the amplification circuit and the data acquisition box. The model of the cutter head used was the FMR04-100-B32-RD16-06, the cemented carbide insert was WIDIA-RDMT1605MOTX, and the workpiece material was the 508III steel in the experiment. The experimental site is shown in Figure 1. According to the common parameters of milling 508III steel in a large number of production practices and scientific experiments, the range of cutting parameters was determined as follows: 188 ≤ *v_c_* ≤ 370 m/min, 0.04 ≤ *f_z_* ≤ 0.08 mm/z, 1 ≤ *a_p_* ≤ 2.5 mm. Where *v_c_* is the cutting speed, *f_z_* is the feed per tooth, and *a_p_* is the axial depth of cut.

The response surface method (RSM) based on statistics is derived from the graph generated after the mathematical model fitting. Experimental data related to the empirical model and the experimental design can be used to fit to describe the research object. Since RSM can analyze the coupling relationship of multiple factors while considering the random error of the experiment, the complex response relationship can be well-fitted based on different order polynomials [20]. Therefore, in order to analyze the influence of different cutting parameters on milling temperature comprehensively and obtain the optimization objective function of milling temperature, the follow-up milling temperature experiment and FE simulation research are carried out based on the Box-Behnken experimental design criteria in the RSM.

### 2.2. Temperature Signal Test of Milling 508III Steel

**Figure 2:**
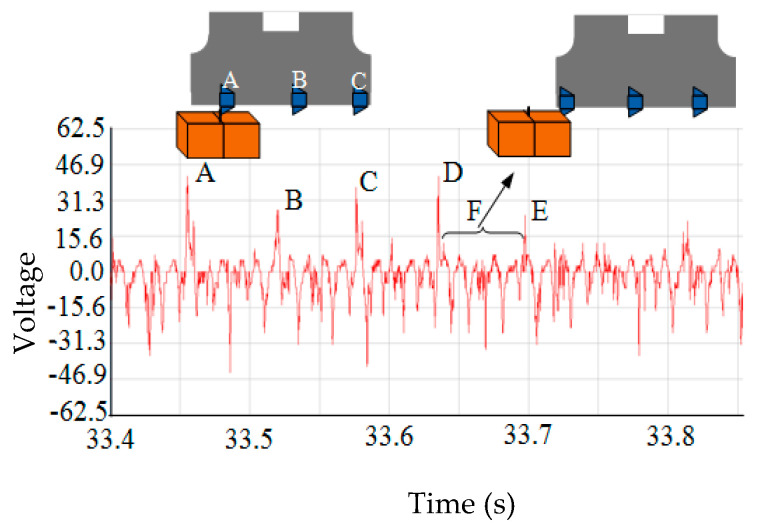
Thermoelectric potential signal diagram of the milling process measured by the experiment.

Figure 2 shows the thermoelectric potential signal measured experimentally. The machining parameters: *v_c_* = 298 m/min, *f_z_* = 0.06 mm/z, and *a_p_* = 2.5 mm.

Due to the soft material of the thermocouple wire, it is difficult to mill and break at one time. Thus, the thermoelectric potential signals (A, B, C, etc.) appeared when cutting to the thermocouple wire. It can be seen from Figure 2 that the intensity of signal E is significantly lower than A, B, C, and D signals. Mainly because after cutting by the front insert, the remaining shorter thermocouple wire is basically insulated from the workpiece. Curve F is the voltage signal between the workpiece and the thermocouple wire.

The time interval between A and B was 33.523 s − 33.466 s = 0.063 s.

The time interval between B and C was 33.582 s − 33.523 s = 0.061 s.

The time interval between C and D was 33.647 s − 33.582 s = 0.065 s.

Due to the problems such as signal interference and measurement equipment response, the voltage signal time interval measured has a certain error. However, the error is small, satisfying the measurement at equal time intervals.

Semi-artificial thermocouples are non-standard thermocouples and need to be calibrated before being used to determine the corresponding relationship between thermoelectric potential and temperature. In this paper, the comparison method of thermocouple indexing [21] was used to obtain the relationship equation between thermoelectric potential and temperature of the semi-artificial thermocouple of the 508III-NiCr.

**Figure 3:**
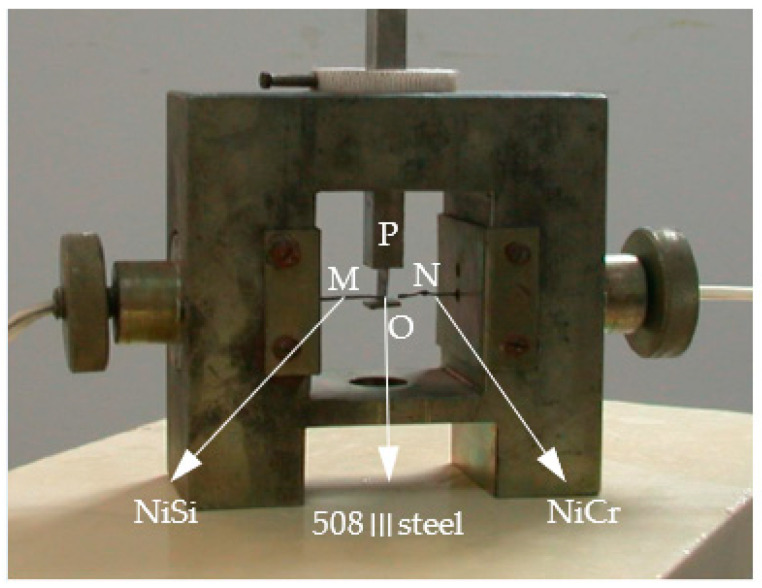
Device diagram of thermocouple indexing calibration.

The main structure of thermocouple indexing calibration is shown in Figure 3. One end of a 508III steel specimen was machined into a thin wall with a thickness of less than 0.5 mm. Meanwhile, one end of the standard nickel silicon wire and nickel-chromium wire was sharpened and pressed against a thin-walled 508III steel test piece with a certain spring pressure. Since the nickel-silicon wire and the nickel-chromium wire were strictly aligned during the operation, the standard nickel-silicon wire, the nickel-chromium wire, and the 508III steel test material were jointly connected to the common node O.

When the nickel-silicon and the nickel-chromium wires formed the temperature calibration through node O, the standard thermocouple MN that gave the temperature signal was used as one input end of the amplifying circuit. When the 508III steel specimen and the nickel-chromium wire passed through node O to form a temperature calibration, the thermocouple to be calibrated gave a temperature signal that was ON, as the other input end of the amplifying circuit. The dynamic signal analyzer was used to collect data from two pairs of thermocouples synchronously, and then the thermoelectric characteristic curve of the 508III-NiCr thermocouple was obtained.

The thermocouple index table of standard NiCr-NiSi at 0 °C is shown in Table 1 [22]. The fitting method for these data can be used to obtain the corresponding relationship between the temperature and electric potential obtained in the experiment. Generally, the voltage curve of the thermocouple is nonlinear, which needs to be corrected when used. The piecewise linear processing method was adopted to correct it in the article.

According to the temperature-thermoelectric potential table of the standard thermocouple of NiCr-NiSi, the quadratic, cubic, and quartic fitting equations of the standard thermocouple can be expressed as:

(1) $$y_{2}=-2.018\times 10^{-6}x^{2}+0.0435x-0.4105$$

(2) $$y_{3}=-4.728\times 10^{-9}x^{3}+7.203\times 10^{-6}x^{2}+0.03872x+0.09695$$

(3) $$y_{4}=-4.65\times 10^{-13}x^{4}-3.519\times 10^{-9}x^{3}+6.195\times 10^{-6}x^{2}+0.03901x+0.07884$$

where *x* is temperature (°C); *y_2_*, *y_3_* and *y_4_* are voltage (mV).

The voltage value of the quadratic fitting equation of the thermocouple has a large error with the standard thermocouple value. However, the error of the voltage value of the cubic and quartic fitting equations is relatively small, and the error value is basically the same. In order to reduce the influence of error accumulation on the accuracy of temperature calculation in the process of indexing and calibration, the semi-artificial thermocouple used in the experiment is also fitted with a cubic equation to obtain its thermoelectric characteristic curve.

**Figure 4:**
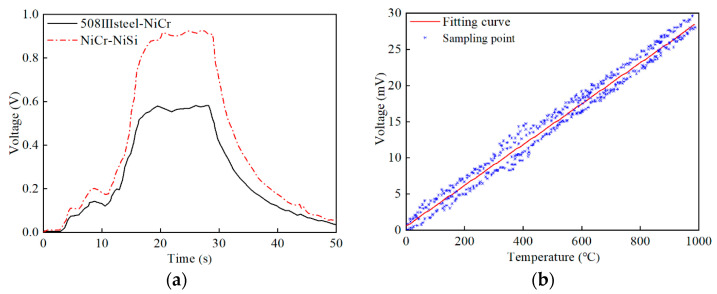
Semi-artificial thermocouple of 508III steel-NiCr. (**a**) Indexing thermoelectric characteristics, (**b**) Fitting curve.

The signals of the standard NiCr-NiSi thermocouple and the 508III steel-NiCr thermocouple to be indexed were collected when amplified by 100 times by the amplifier circuit. The sampling frequency was 1000 Hz. The voltage signal collected during the process of indexing and calibrating the 508III steel-NiCr semi-manual thermocouple is shown in Figure 4a. It can be seen that the voltage change trends of the two thermocouples are basically the same, indicating that the temperature felt is basically the same at the connection point of the two thermocouples. It can satisfy the basic conditions of the comparison method for indexing and calibrating thermocouples and ensure the accuracy and reliability of the calibration results.

By repeating indexing and calibration experiments, the fitting curve of the 508III steel-NiCr semi-artificial thermocouple obtained is shown in Figure 4b. The fitting equation of the 508III steel-NiCr semi-artificial thermocouple is shown in Equation (4). From Figure 4b, it can be found that the sampling points are basically evenly distributed on both sides of the fitting equation curve, and the overall linearity of the fitting curve is good, which can provide an effective way for the experimental measurement of the cutting temperature of 508III steel.

(4) $$y=1.12155\times 10^{-9}x^{3}-1.84643\times 10^{-6}x^{2}+0.029072086x+0.50059124$$

where *x* is temperature (°C); *y* is voltage (mV).

### 2.3. Simulation Analysis of the Temperature

**Figure 5:**
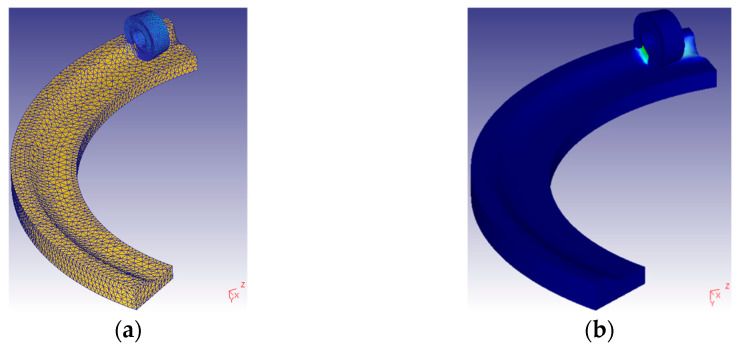
FE simulation. (**a**) FE models of the insert and workpiece, (**b**) Simulation process.

The DEFORM-3D simulation software was adopted to simulate and explore the changes in temperature under given cutting parameters during the milling of 508III steel. In this study, the maximum value of the simulation temperature, which is approximately stable, is used as the value of the milling temperature. FE models of the insert and the workpiece are shown in Figure 5a. A simulation of the milling process is depicted in Figure 5b. Some common performance parameters of tool and workpiece materials are shown in Table 2.

The number of tool and workpiece model elements is 30,000 and 40,000, respectively. The Johnson-Cook constitutive equation was selected as the original construction model, and its expression is shown in Equation (5).

(5) $$\bar{\sigma }=\left(A+B{\bar{\varepsilon }}^{n}\right)\left(1+C\ln\frac{\dot{\bar{\varepsilon }}}{{\dot{\bar{\varepsilon }}}_{0}}\right)\left[1-{\left(\frac{T-T_{\mathrm{room}\;}}{T_{\mathrm{melt}}-T_{\mathrm{room}}}\right)}^{m}\right]$$

where *A, B, C*, m, and *n* are the material constants of the Johnson-Cook constitutive equation, *T* is the local temperature, *T_room_* is the room temperature, *T*_melt_ is the melting temperature, $\bar{\varepsilon }$ is the equivalent plastic strain, $\dot{\bar{\varepsilon }}$ is the equivalent strain rate, and ${\dot{\bar{\varepsilon }}}_{0}$ is the reference value of the strain rate. The model constants adopted are listed in Table 3.

## 3. Results and Discussion

### 3.1. Simulated and Experimental Results

**Figure 6:**
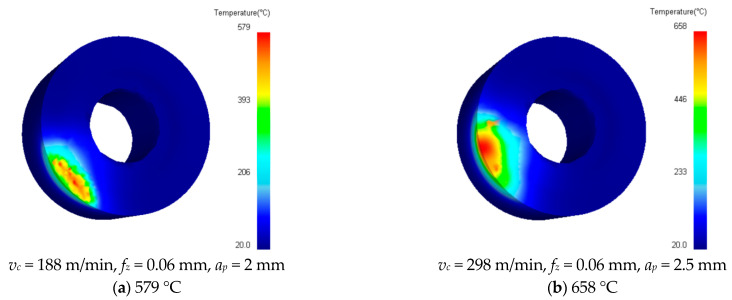

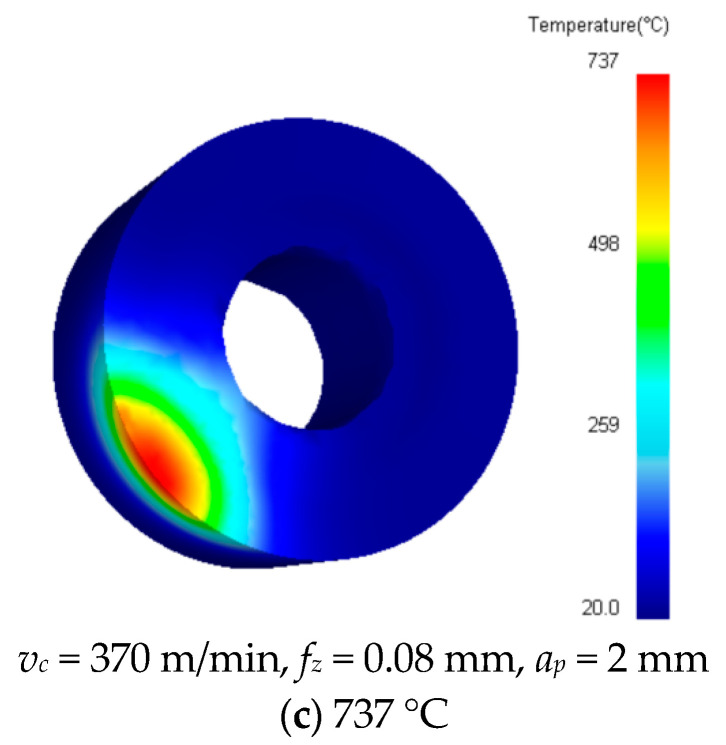
Simulated cloud diagram of milling temperature.

This section gives some temperature simulation cloud diagrams with different combinations of cutting parameters in Table 4, as shown in Figure 6. The temperature simulation cloud pictures under other cutting parameter combinations are no longer listed individually.

It can be observed from Figure 6 that the temperature of the area near the main cutting edge of the rake face of the milling insert is the highest, and the temperature away from the main cutting edge of the rake face is lower. The main reason is that the area is the actual cutting part during the milling process. Due to the high pressure and severe friction at this place, a large amount of frictional heat is generated in contact with the chip, and the heat is concentrated, resulting in the high temperature in this area. Other parts not involved in cutting would produce a temperature effect under the heat transfer of blade material, resulting in a decrease in temperature away from the main cutting edge.

The comparison results of milling temperature experiment data and FE simulation are shown in Table 4. The absolute value of the relative error calculated is within 5%. Results show that the FE analysis can accurately simulate the milling process of 508III steel, and simulation data have a certain validity. Meanwhile, it can also provide reliable data for the following temperature prediction, parameter optimization, and validity verification research.

### 3.2. Influence of Cutting Parameters Interaction on Milling Temperature

According to the experimental results of milling temperature in Table 4, the influence of the interaction was analyzed between cutting parameters on the milling temperature based on RSM.

**Figure 7:**
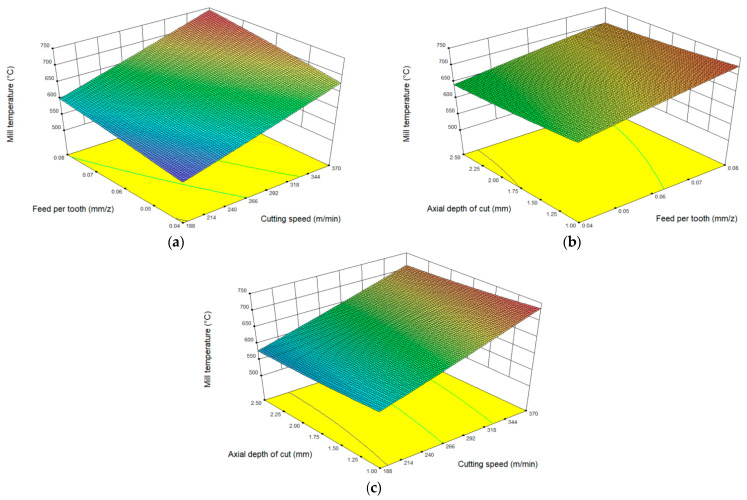
Response surface of interaction between different cutting parameters to milling temperature. (**a**) Influence of feed per tooth and cutting speed on milling temperature, (**b**) Influence of axial depth of cut and feed per tooth on milling temperature, (**c**) Influence of axial depth of cut and cutting speed on milling temperature.

The response surface of the influence of feed rate per tooth and cutting speed on milling temperature is shown in Figure 7a. It can be obtained that with the increase in cutting speed and feed rate per tooth, the milling temperature shows an obvious upward trend. The main reason is that with the increase in cutting speed and feed rate per tooth, the amount of metal removal per unit of time increases, resulting in greater heat in the milling area, and less heat is taken away by chips. Thus, the milling temperature increases. The combination of a smaller feed per tooth and a lower cutting speed can reduce the milling temperature of the tool.

The response characteristics of the temperature to the combination of axial depth of cut and feed per tooth and the combination of axial depth of cut and cutting speed are, respectively, shown in Figure 7b,c. It can be found that with the increase in the axial cutting depth, the range of milling temperature change is small and accompanied by a decreasing trend. The main reason could be that as the axial depth of the cut increases, the contact area between the tool and the workpiece increases, resulting in more heat taken away by the workpiece and chip, so that the cutting temperature has a downward trend.

### 3.3. Construction of Mathematical Model for Temperature Prediction

In actual machining, temperature prediction is of great significance for improving tool life and ensuring product quality. Cutting parameters are one of the main factors affecting milling temperature. Thus, based on the above temperature experimental results and polynomial fitting method in the RSM, the temperature prediction mathematical model is studied with cutting speed, feed per tooth, and axial depth of cut as independent variables and temperature as dependent variables.

In the RSM, the regression prediction model is usually built based on a second-order polynomial. Therefore, in order to analyze the response characteristics of milling temperature to process parameters, a quadratic polynomial regression prediction model for milling temperature is established in this paper. Its expression is shown in Equation (6).

(6) $$T=g_{0}+\sum_{i=1}^{3}g_{i}\cdot x_{i}+\sum_{i=1}^{3}\sum_{j=1}^{3}g_{ij}\cdot x_{i}\cdot x_{j}$$

where *g_0_* is the initial undetermined value; *g_i_* is the influence coefficient of *x_i_*; *g_ij_* is the interaction influence coefficient of *x_i_* and *x_j_*; *x_1_, x_2_*, and *x_3,_* respectively, represent the cutting speed, feed per tooth, and axial cutting depth.

Multivariate regression fitting is conducted according to the experimental temperature results in Table 4. The temperature quadratic polynomial regression prediction model is constructed, as shown in Equation (7).

(7) $$\begin{array}{l} T=377.65379+0.62898v_{c}+1838.37595f_{z}-21.63608a_{p}+\\ \;1.20172v_{c}f_{z}-0.031719v_{c}a_{p}-59.58154f_{z}a_{p}+\\ \;1.76668\times 10^{-4}v_{c}{}^{2}-4976.34313f_{z}{}^{2}+6.31138a_{p}{}^{2} \end{array}$$

### 3.4. Significance Test of Temperature Model

In order to determine the degree of correlation between temperature variation characteristics and cutting parameters, it is necessary to conduct a significance test on the temperature regression model. The model significance can be determined through variance analysis and significance tests [23]. Therefore, the *F*-test method was adopted for model significance verification in this paper. The results of the temperature regression model significance test based on this method are shown in Table 5.

From Table 5, it can be found that the *F* statistic value of the regression model is 110.20, which is far greater than the value of *F*_0_._05_(9, 7) = 3.68 in the quantile table of *F* distribution. Results show that the regression model constructed has a high significance under 95% confidence.

### 3.5. Mathematical Model of Cutting Efficiency

Based on metal cutting theory, cutting efficiency can be measured by material removal rate, and the mathematical model is shown in Equation (8).

(8) $$Q=v_{f}\cdot a_{p}\cdot a_{e}=\frac{1000z}{\pi d}v_{c}\cdot f_{z}\cdot a_{p}\cdot a_{e}$$

where: *z* represents the number of cutter teeth involved in machining, *d* represents the cutter diameter, and $a_{e}$ is a fixed value of 50 mm.

The mathematical models of temperature prediction and cutting efficiency established have good effectiveness and reliability as the objective function of cutting parameter optimization.

## 4. Temperature Prediction Based on SVM

### 4.1. GA-Optimized SVM Model

The SVM has strong generalization performance under the condition of a small sample dataset, which is a powerful machine learning model and more suitable for nonlinear regression problems [24]. Due to the nonlinearity and limited milling temperature data, SVM was used for temperature prediction. In this study, the radial basis function was used as the SVM kernel function, and its form is shown in Equation (9).

(9) $$K\left(X_{i},X_{j}\right)=\exp\left(-\frac{1}{2}{\left(\frac{\left\|X_{i}-X_{j}\right\|}{\sigma }\right)}^{2}\right)$$

**Figure 8:**
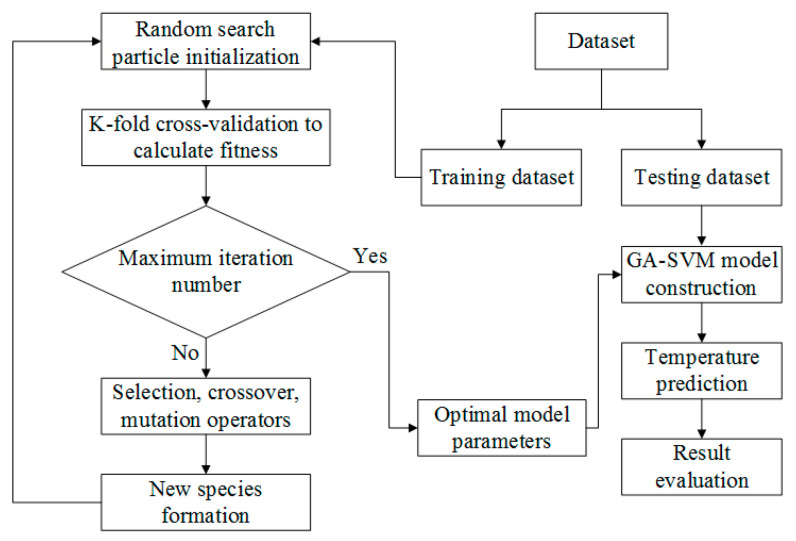
Flow diagram of the GA-SVM.

The prediction performance of the SVM model depends on the super parameters of SVM strongly: penalty parameter *C* and kernel parameter *σ* [25]. In order to further improve the prediction accuracy of SVM, a genetic algorithm was used to optimize the super parameters of SVM. The prediction model of SVM optimized by the genetic algorithm was established, and its basic process is shown in Figure 8.

### 4.2. Temperature Prediction Based on GA-SVM

MATLAB and libsvm3.24 program packages were adopted to compile the regression program, and different cutting parameter combinations were used as the input of the GA-SVM model to predict the change in milling temperature. Temperature data obtained by simulation were used in the training set, and the 5-fold cross-validation was carried out using the experimental data as the test set.

In this study, the maximum iteration was used as the termination standard of the algorithm, and the average value of the mean square error of the output results of the 5-fold cross-validation was used as the fitness of the search particle in the optimization process. Therefore, the search particle corresponding to the minimum fitness function value was the ideal model parameter of SVM. The initial range of SVM super parameters: *C* ∈ [10^−4^,10^4^], *σ* ∈ [10^−4^,10^4^]. GA algorithm: population size is 20, maximum iteration is 200, and individual selection probability is 0.9.

**Figure 9:**
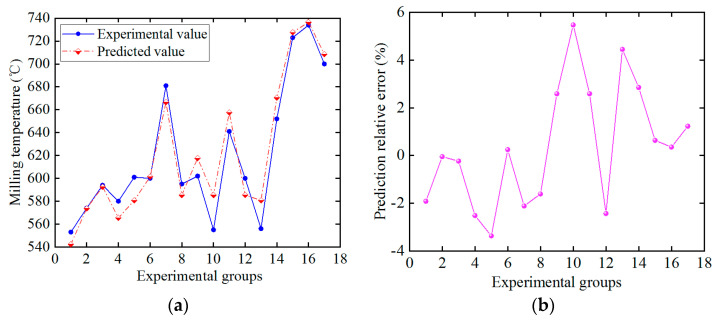
Temperature prediction. (**a**) Comparison of experimental and predicted value, (**b**) Relative error.

Regression prediction of milling temperature was carried out based on the GA-SVM model, and the prediction results are shown in Figure 9a. It can be found that the predicted value of the GA-SVM model is basically consistent with the changing trend of temperature experiment results. Figure 9b shows the relative error of temperature predicted by the GA-SVM model. The error range is −3.37%~5.48% and the absolute value of the maximum relative error is within 6%, indicating that the temperature prediction method based on the GA-SVM model has certain effectiveness and reliability.

## 5. Cutting Parameter Optimization Based on NSGA-II Algorithm

### 5.1. NSGA-II Algorithm

**Figure 10:**
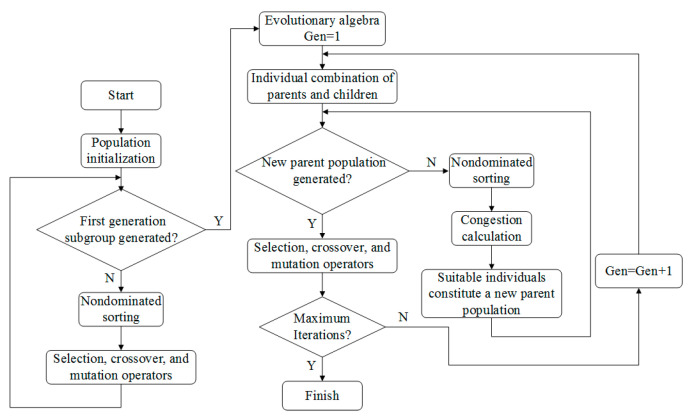
Flow diagram of the NSGA-II.

Elitist nondominated sorting genetic algorithm (NSGA-II) is widely used due to its strong robustness and convergence, as well as high computational efficiency and good convergence for multi-objective optimization problems with two or three objectives [26]. Thus, the NSGA-II algorithm is used to study the optimization of cutting parameters. The specific process is shown in Figure 10.

### 5.2. Construction of Optimization Objective Function and Constraint Conditions

Since 508III steel is difficult to machine, cutting parameter optimization is very important to reduce cutting temperature and improve tool life and machining efficiency. Therefore, this study optimizes the cutting parameters based on the NSGA-II algorithm with milling temperature as the optimization objective function. The cutting parameter optimization problem in this paper is to solve the minimum value of the objective function of cutting temperature and the maximum value of the objective function of cutting efficiency. Thus, the form of the optimization objective function is shown in Equation (10).

(10) $$\underset{x\in R}{\min}=\left(T\left(v_{c},\;f_{z},\;a_{p}\right),-Q\left(v_{c},\;f_{z},\;a_{p}\right)\right)$$

It is important to constrain the influencing factors of the optimization objective function to meet the conditions of the milling process of 508III steel and make it meaningful to optimize the objective function to optimize the cutting parameters. Since this paper mainly studies the effect of cutting parameters on cutting temperature and cutting efficiency, the range of cutting parameters is limited. The set constraints are as follows:

(1) Cutting speed constraints:
  (11) $$v_{c}=188\sim 370\;m/\min$$
(2) Constraints of feed rate per tooth:
  (12) $$f_{z}=0.04\sim 0.08\;\mathrm{mm}/z$$
(3) Constraints on axial cutting depth:
  (13) $$a_{p}=1\sim 2.5\;\mathrm{mm}$$

### 5.3. Parameter Optimization Results and Discussion

Based on the cutting temperature and efficiency mathematical model constructed in Section 3 and the process of NSGA-II optimization in Section 5.1 above, the optimal combination of the cutting parameter is obtained by MATLAB programming. During the cutting parameter optimization using the NSGA-II algorithm, the specific settings of relevant parameters of the NSGA-II algorithm are as follows: population size = 50, maximum iteration = 200, individual selection probability = 0.9, and mutation probability = 0.05.

**Figure 11:**
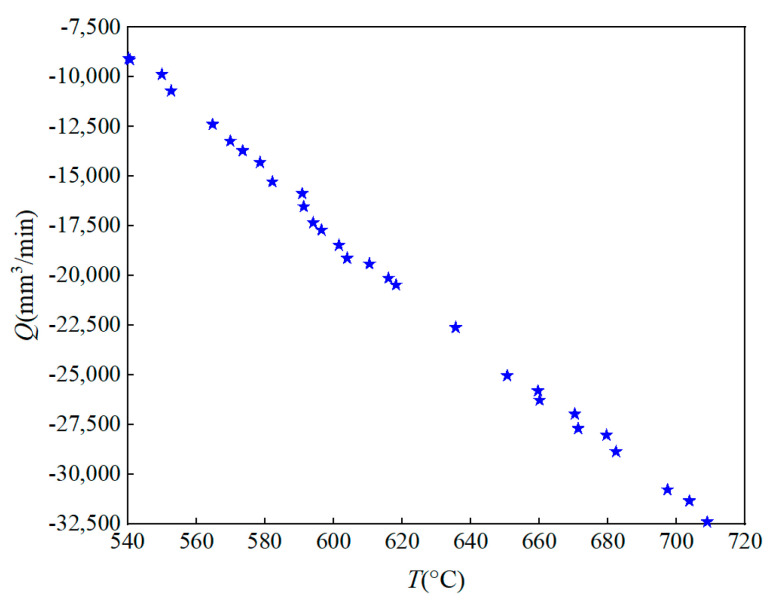
Pareto optimal frontier.

Due to the randomness of the parameter optimization of the swarm intelligence optimization algorithm, optimized programs were run 25 times. The Pareto optimal frontier of the two optimization objectives is obtained for the 25th optimization, as shown in Figure 11. It can be found that the cutting efficiency increase with the increase in cutting temperature. There is a conflict between cutting efficiency and temperature. By analyzing the multi-objective optimization function, 30 groups of Pareto optimal solutions are obtained. Some Pareto optimization solution set results are shown in Table 6. Decision-makers can reasonably select processing parameters based on actual processing needs, effectively improving decision−making efficiency.

## 6. Conclusions

In this paper, the milling temperature of cemented carbide tool in machining 508III steel is studied based on the temperature experiment, FE simulation, GA-SVM prediction, and multi-objective parameter optimization. Some main conclusions can be drawn as follows:

(1) Based on the Box-Behnken experimental design criteria in RSM, a series of milling temperature experiments and FE simulations were carried out. The temperature was measured by using the semi-artificial thermocouple fitting equation of 508III steel-NiCr effectively.
(2) Based on the FE simulation results, it was found that the area near the main cutting edge of the rake face produced a lot of friction heat in contact with the chip due to high pressure and serious friction, resulting in a higher temperature. By comparing the temperature experiment with the FE results, it was found that the absolute value of the relative error was within 5%. Results showed that the simulation results were accurate and effective, which verified the reliability of the FE analysis method and provided data support for the validation of parameter optimization effectiveness.
(3) On an experimental basis, the influence of the interaction of cutting parameters on the temperature was analyzed based on RSM. It was found that the temperature increased significantly with the increase in cutting speed and feed rate per tooth, and the temperature changed less with the increase in axial cutting depth, accompanied by a decreasing trend. Based on RSM, a second-order polynomial prediction model for temperature was constructed. The variance and F-test results showed that the temperature regression model constructed had high significance at a 95% confidence level and good effectiveness and reliability.
(4) The SVM method was used for temperature prediction, and the GA-SVM model was established to predict cutting temperature. It was found that the prediction error range was −3.37~5.48%, indicating that the method had certain effectiveness and reliability.
(5) Taking cutting temperature and efficiency as evaluation indicators, the NSGA-II algorithm was used to optimize cutting parameters. The Pareto optimal frontier and 30 groups of Pareto optimal solutions were obtained, and Decision makers can reasonably select processing parameters based on actual processing needs, effectively improving decision-making efficiency.

## Figures and Tables

**Table 1:** Comparison of fitting equations of the standard thermocouple.

Temperature (°C)	Quadratic Fitting Voltage Value (mV)	Cubic Fitting Voltage Value (mV)	Quartic Fitting Voltage Value (mV)	Standard Voltage Value (mV)	Quadratic Fitting Relative Error (%)	Cubic Fitting Relative Error (%)	Quartic Fitting Relative Error (%)
100	3.9193	4.0363	4.0382	4.0950	−4.2906	−1.4543	−1.3871
200	8.2088	8.0912	8.0997	8.1370	0.8824	−0.5661	−0.4584
300	12.4579	12.2336	12.2406	12.2070	2.0554	0.2174	0.2753
400	16.6666	16.4348	16.4369	16.3950	1.6566	0.2422	0.2556
500	20.8350	20.6667	20.6637	20.6400	0.9448	0.1292	0.1148
600	24.9630	24.9008	24.8947	24.9020	0.2450	−0.0048	−0.0293
700	29.0507	29.1087	29.1027	29.1280	−0.2654	−0.0663	−0.0869
800	33.098	33.2621	33.2594	33.2770	−0.5379	−0.0448	−0.0529
900	37.1049	37.3327	37.3354	37.3250	−0.5897	0.0206	0.0279
1000	41.0715	41.292	41.2998	41.2690	−0.4786	0.0557	0.0746
1100	44.9977	45.1116	45.1212	45.1080	−0.2445	0.0080	0.0293
1200	48.8836	48.7633	48.7666	48.8280	0.1139	−0.1327	−0.1258
1300	52.7291	52.2186	52.2021	52.3930	0.6415	−0.3340	−0.3644

**Table 2:** Parameters of tool and workpiece materials.

Name	Cemented Carbide	508III Steel
Elastic modulus (GPa)	600	212
Density (g/cm^3^)	14.5	7.9
Thermal conductivity (W/(m·K))	62.8	14.5
Specific heat capacity (kg·K)	460	460
Poisson’s ratio	0.33	0.3

**Table 3:** Relevant parameters of the J-C model.

Parameters	Value
*A*	1766
*B*	904
*C*	0.001
*n*	0.144
*m*	0.72
${\dot{\bar{\varepsilon }}}_{0}$	1
*T_room_* (°C)	20
*T_melt_* (°C)	1650

**Table 4:** Simulated and experimental results.

Exp. No.	Cutting Speed *v_c_* (m/min)	Feed Per Tooth *f_z_* (mm/z)	Axial Depth of Cut *a_p_* (mm)	Milling Temperature (°C)		
				Experimental Results	Simulated Results	Relative Error
1	298	0.06	2	656	661	−0.76%
2	370	0.06	1	723	728	−0.69%
3	298	0.08	2.5	677	693	−2.31%
4	298	0.04	2.5	620	610	1.64%
5	370	0.06	2.5	697	706	−1.27%
6	298	0.08	1	693	678	2.21%
7	188	0.08	2	593	578	2.66%
8	298	0.06	1	668	686	−2.62%
9	188	0.04	1	553	542	2.03%
10	188	0.06	2	574	579	−0.86%
11	298	0.06	2.5	641	658	−2.58%
12	370	0.04	2.5	672	692	−2.89%
13	298	0.04	2	611	620	−1.45%
14	298	0.08	2	681	659	3.34%
15	188	0.08	2.5	594	593	0.17%
16	298	0.04	1	634	651	−2.61%
17	370	0.08	2	734	737	−0.41%

**Table 5:** Temperature regression model significance test.

Variance Analysis	DF	SS	MS	F
Regression	9	43,038.49	4782.05	154.2
Residual	7	217.04	31.01	-
Total	16	43,255.53	-	-

**Table 6:** Pareto optimization solution set.

Number	$v_{c}$ (m/min)	$f_{z}$ (mm/z)	$a_{p}\;(\mathrm{mm})$	*T* (°C)	$Q({\mathrm{mm}}^{3}/\min)$
A1	188.08541	0.0406	2.48802	540.29024	−9070.72647
A2	191.21765	0.0583	2.48442	569.94189	−13,223.27547
A3	251.56217	0.07634	2.46614	635.65477	−22,611.9388
A4	203.50428	0.07651	2.48388	601.58856	−18,465.67747
A5	201.09502	0.06932	2.48247	591.35964	−16,522.64736
A6	295.59058	0.07904	2.48303	671.34076	−27,698.27075
A7	268.83171	0.07841	2.48765	650.66639	−25,037.65621
A8	191.76944	0.05446	2.48339	564.76379	−12,383.03666
⋮	⋮	⋮	⋮	⋮	⋮

## Data Availability

The data presented in this study are available from the corresponding author upon reasonable request.
